# Characterization of vaginal microbiota in women with preterm labor with intra-amniotic inflammation

**DOI:** 10.1038/s41598-019-55611-y

**Published:** 2019-12-12

**Authors:** Teresa Cobo, Andrea Vergara, Maria Carmen Collado, Climent Casals-Pascual, Eduardo Herreros, Jordi Bosch, Ana B. Sánchez-García, Rosa López-Parellada, Júlia Ponce, Eduard Gratacós

**Affiliations:** 1 BCNatal - Barcelona Center for Maternal-Fetal and Neonatal Medicine (Hospital Clínic and Hospital Sant Joan de Deu), Fetal i+D Fetal Medicine Research Center, IDIBAPS, University of Barcelona, Barcelona, Spain; 2 Centre for Biomedical Research on Rare Diseases (CIBER-ER), Barcelona, Spain; 3 Microbiology, Biomedical Diagnostic Center, Hospital Clínic and ISGlobal (Barcelona Institute for Global Health), University of Barcelona, Barcelona, Spain; 4 Department of Biotechnology, Institute of Agrochemistry and Food Technology-Spanish National Research Council (IATA-CSIC), Paterna, Valencia, Spain

**Keywords:** Bacterial genes, Predictive markers

## Abstract

This study aims to investigate the relation between vaginal microbiota and exposition to intra-amniotic inflammation (IAI). We conducted a prospective cohort study in women with preterm labor <34 weeks who had undergone amniocentesis to rule out IAI. Vaginal samples were collected after amniocentesis. Women with IAI included those with positive amniotic fluid (AF) for a microorganism identified by specific culture media and Sanger sequencing 16S ribosomal RNA gene and/or high AF interleukin (IL)-6 levels. Vaginal microbiota was characterized by 16S ribosomal RNA gene amplicon sequencing. Specific quantitative PCR targeted to Lactobacillus spp. was also performed. Regression models were used to evaluate associations between vaginal microbiota and exposition to IAI. Concerning our results, 64 women were included. We observed an inverse association between AF IL-6 levels and load of Lactobacillus spp. Depletion in Lactobacillus spp. load was significantly associated with an early gestational age at delivery and a short latency to delivery. Microbial-diversity was found to be a risk factor for the subsequent occurrence of clinical chorioamnionitis. To the contrary, higher Lactobacillus spp. load had a protective role. In conclusion, the study identifies reduced bacterial load of Lactobacillus spp. in women exposed to IAI and found microbial-diversity and Lactobacillus spp. depletion to be associated with a worse perinatal outcome.

## Introduction

The effects of prematurity on infants, parents and society make preterm birth an important issue in public health worldwide^1^. The time of presentation of symptoms is important in the pathophysiology of preterm labor (PTL)^2,3^. Therefore, the earliest spontaneous preterm births (sPTB)^4^ are most likely considered related to intra-amniotic inflammation (IAI).

IAI can develop through microbial invasion of the amniotic cavity (MIAC) or through other mechanisms related to endogenous mediators from the placenta and fetal membranes that trigger IAI similarly to MIAC but leading to “sterile” IAI^5^.

The use of next-generation sequencing techniques has been instrumental to describe and characterize the healthy human vaginal environment. The depletion of *Lactobacillus* spp. has been related to a poor reproductive^6^ and infectious outcome^7–10^.

Little is known on the relation between vaginal microbiota composition and exposition to IAI in women with PTL. Concerning this, Hitti *et al*.^11^ did observe a high expression of vaginal cytokines, an abnormal vaginal Gram stain, absence of hydrogen peroxide-producing Lactobacillus and the presence of anaerobic vaginal flora in the vaginal cultures of women with MIAC and IAI. However, this standard microbiological method of diagnosis only identifies a small part of the multitude of microorganisms, many of which are very difficult to cultivate, or are considered non-cultivable.

In this scenario, the main aim of this study was to evaluate the relation between vaginal microbiota and exposition to IAI in women with PTL determined by 16S ribosomal RNA gene amplicon sequencing and specific quantitative PCR targeted to *Lactobacillus* spp. We also aimed to investigate the association between vaginal microbiota and gestational age at delivery and other infectious/inflammatory outcomes.

## Results

### Baseline characteristics of the study population

Sixty-nine women were included. Maternal characteristics and pregnancy management are summarized in Table 1. Data of delivery was missing in 5 women with No-MIAC/Non-IAI.

**Table 1:** Maternal characteristics and pregnancy outcomes.

	IAI (n = 21)	No-MIAC/Non-IAI (n = 43)	*p*
Maternal age (y)	32.0 (26.8; 36.4)	33.2 (28.8; 38.3)	0.548
Body Mass Index (kg/m^2^)	22.2 (20.5; 25.8)	22.3 (20.9; 25.5)	0.966
Ethnicity			0.287
-Caucasian	13 (62)	31 (72)	
-Afro-Caribbean	2 (10)	1 (2)	
-Arabian	5 (24)	7 (16)	
-Black	0	2 (5)	
-Asian	1 (5)	0	
-Other	0	2 (5)	
Nulliparity	14 (67)	23 (53)	0.316
Smoking	3 (14)	3 (7)	0.346
Diabetes	0	1 (2)	0.481
Gestational age at admission (w)	25.7 (23.2; 28.1)	29.7 (25.9; 31.6)	0.001
Gestational age at vaginal sampling (w)	25.7 (23.3; 28.3)	29.9 (26.4; 31.7)	0.001
CRP (mg/dL) at admission	2.1 (0.9; 4.0)	0.5 (0.3; 1.1)	<0.001
WBC count (×10^9^) at admission	13580 (10730; 15295)	12280 (9010; 15200)	0.268
Amniotic fluid IL-6 (ng/mL)	51.6 (23.4; 187.3)	1.2 (0.7; 2.7)	<0.001
Cervical length (mm) at admission	8.0 (0; 16.5)	11.0 (6.0; 22.0)	0.128
Antibiotics prior to vaginal sampling	10 (48)	9 (21)	0.028
Latency from antibiotic administration to vaginal sampling (d)	0.5 (0; 1)	0.5 (0; 2)	0.652
Steroids prior to vaginal sampling	9 (43)	32 (74)	0.013
Tocolysis during lung maturation	15 (71)	41 (95)	0.012
Cerclage prior to vaginal sampling	3 (14)	2 (5)	0.177
Latency from sampling to delivery (d)	4.0 (1.0; 12.5)	42.0 (19.0; 67.8)	<0.001
Gestational age at delivery (w)	27.6 (25.6; 30.4)	36.8 (32.4; 38.4)	<0.001
- Spontaneous delivery <28.0 w	11/15 (73)	0	<0.001
- Spontaneous delivery <32.0 w	19/20 (95)	8/32 (25)	<0.001
- Spontaneous delivery <34.0 w	19 (90)	13/38 (34)	<0.001
- Spontaneous delivery <37 w	20 (95)	19/38 (50)	<0.001
Clinical chorioamnionitis at the onset of labor	6 (29)	1/38 (3)	0.003

Two vaginal samples were excluded from the analyses due to the low number of reads obtained and three due to the presence of high levels of *Burkholderia cepacia*, considered external contaminant bacteria.

The final sub-set (n = 64) included 14 women with MIAC.

The most common microorganism identified in the amniotic fluid was *Ureaplasma* spp. (n = 5). Others were *Mycoplasma hominis* (n = 1), *Mycoplasma hominis* and *Fusobacterium nucleatum* (n = 1), *Sneathia sanguinegens* and *Prevotella amnii* (n = 1), *Streptococcus agalactiae* (n = 1), *Streptococcus lutetiensis* (n = 1), *Capnocytophaga sputigena* (n = 1), *Candida albicans* (n = 1), *Roseomonas mucosa* (n = 1), *Rhodococcus* spp. (n = 1). The only microorganisms identified by Sanger sequencing 16S ribosomal RNA but not by culture were Roseomonas mucosa and Rhodococcus spp. Both presented high levels of amniotic fluid IL-6. The other microorganisms were identified by culture and confirmed by Sanger sequencing of the 16S ribosomal RNA gene.

### Alpha and Beta-diversity of the vaginal microbiota in women with IAI

**Figure 1:**
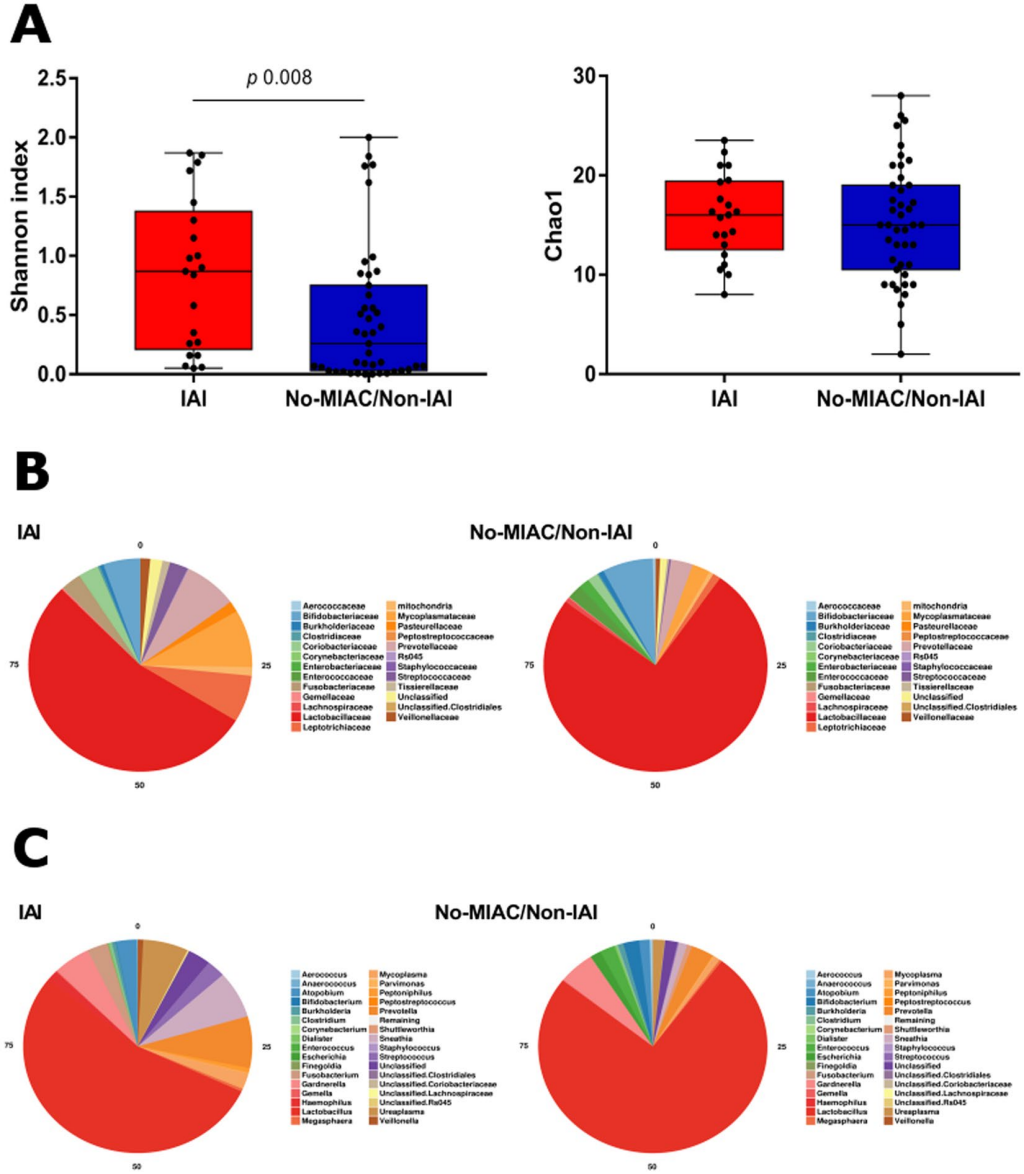
(**A**) Boxplot showing alpha diversity metrics of Shannon index (microbial-diversity) and Chao1 (richness) in women with or without IAI. (**B**) Comparison and relative abundance of microbial taxa at family level in women with or without IAI. (**C**) Comparison and relative abundance of microbial taxa at genus level in women with or without IAI.

Women with IAI had higher microbial-diversity (*p* = 0.008, Shannon index) but not higher richness than the No-MIAC/Non-IAI group (Fig. 1A).

Beta-diversity analysis of microbial communities using both weighted and unweighted UNIFRAC and, also, Bray Curtis distance did not show differences in clustering by IAI (Supplemental data S1).

### Vaginal microbiota composition in women with IAI

Differences in the relative abundance of bacteria have been observed at family (Fig. 1B) and genus level (Fig. 1C). A higher relative abundance of *Lactobacillus* genus was observed in women with No-MIAC/Non-IAI and a higher microbial-diversity in women with IAI.

**Figure 2:**
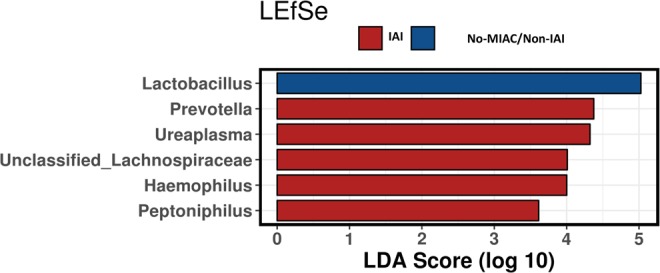
LEfSe test at genus level.

Using the LEfSe test at the genus level, we found enrichment of *Ureaplasma* (LDA 4.34 score, *p* = 0.008), *Peptoniphilus* (LDA 3.73 score, *p* = 0.050), *Gardnerella* (LDA 4.56 score, *p* = 0.039) and *Haemophilus* (LDA 4.37 score, *p* = 0.077) in women with IAI and of *Lactobacillus* (LDA 5.09, *p* = 0.038) in women with No-MIAC/Non-IAI (Fig. 2).

**Figure 3:**
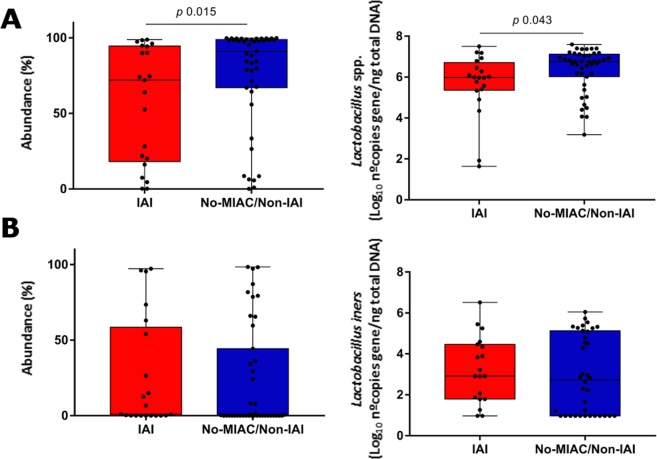
Lactobacillus spp. (**A**) and L. iners (**B**) relative abundance (%) by sequencing and total load by specific quantitative PCR.

Similar observation on *Lactobacillus* genus abundance was observed by both methodologies, sequencing as relative abundance (*p* = 0.015) and specific qPCR as total load *(p* = 0.043). We found a depletion of *Lactobacillus* spp. load in women with IAI but not of *Lactobacilllus iners* (Fig. 3).

### Correlation between microorganisms found in the AF and vaginal microbiota

**Figure 4:**
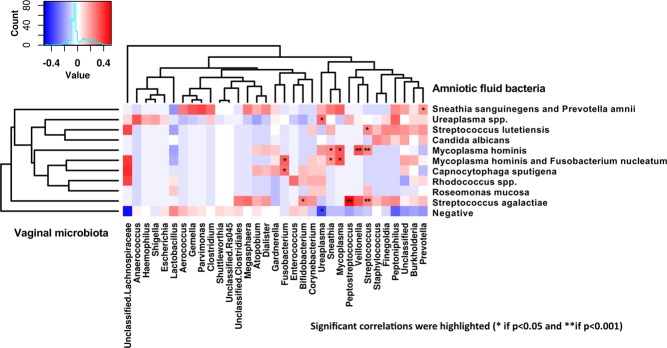
Spearman correlation analysis between amniotic fluid and vaginal microbiota.

Correlation between microorganisms isolated in the AF and vaginal microbiota was performed using Spearman correlation analysis (Fig. 4). Most of the microorganisms isolated in the AF were also found in the vaginal microbiota except for *Roseomonas mucosa, Rhodococcus* spp. *and Capnocytophaga sputigena*.

### Association between IAI and vaginal microbiota

We observed a significant inverse association between IL-6 concentrations and load of *Lactobacillus* spp. (β −0.253, t −2.76, p 0.008).

No association was found between microbial-diversity and IL-6 (β 0.334, t 1.77), p 0.081).

### Vaginal microbiota and infectious outcomes

Microbial-diversity (Shannon index) was found to be a risk factor (OR 4.1 95% CI 1.18–14.09) for the subsequent occurrence of clinical chorioamnionitis. To the contrary, *Lactobacillus* spp. load had a protective role (OR of 0.5 95% CI 0.29–0.88).

### Vaginal microbiota, gestational age at delivery and spontaneous preterm birth

*Lactobacillus* spp. load depletion was significantly associated with an early gestational age at delivery (β 0.359, t (57) 2.742, p 0.008) and a short latency from vaginal sampling to delivery (β 0.29, 2.151 (57), p 0.036). To the contrary, in the binary logistic regression model, the *Lactobacillus* spp. load was found to be a protective factor for the occurrence of spontaneous delivery within 7 days (OR of 0.34 95% CI 0.17–0.68) and <34 weeks (OR 0.55 95% CI 0.31–0.98). The ROC curve showed an area under the curve of load of *Lactobacillus* spp. for predicting sPTB <34 weeks of 0.69, with a 95% confidence interval of 0.55–0.83. No associations between *Lactobacillus iners* and the different outcomes evaluated were observed.

## Discussion

Our study showed that total *Lactobacillus* genus load is inversely associated with IAI. In agreement with previous studies^13–15^, *Lactobacillus* spp. depletion is associated with early gestational age at delivery and short latency from vaginal sampling to delivery. Furthermore, both microbial-diversity and *Lactobacillus* spp. depletion are associated with subsequent clinical chorioamnionitis.

It is well known that the prevalence of IAI is of up to 40% when symptoms of PTL occur <28 weeks^2–4^. For clinicians, the importance to identify this inflammatory condition is to select the group of women with the highest risk of delivery within the following days^16^.

Despite the clinical relevance of IAI, amniocentesis is still required for achieving diagnosis at present. This has become an incentive to explore the intra-amniotic environment through alternative and non-invasive strategies such as the characterization of the vaginal or the cervical fluid microbiota composition.

*Lactobacillus* has been proposed as one of the vaginal microbes that modulate maternal response to infection and down-regulates inflammation^7,8,10^ inhibiting induction of pro-inflammatory cytokines^17^. Concerning pregnancy, there is increasing evidence showing that non-Lactobacillus bacteria generally associated with bacterial vaginosis are highly correlated with the expression of pro-inflammatory cytokines, which may play a role in the onset of labor in women with sPTB^10,18^. Moreover, there are some trials suggesting a reduction in the recurrence of bacterial vaginosis in women treated with Lactobacillus^19^.

What is of note in our paper is that it characterizes vaginal microbiota composition in women with PTL who had undergone amniocentesis to rule out IAI. In this regard, Hitti *et al*.^11^ did observe a high expression of vaginal cytokines, an abnormal vaginal Gram stain, absence of hydrogen peroxide-producing Lactobacillus and the presence of anaerobic microorganisms in the vaginal cultures of women with MIAC and IAI. In line with these authors, we also found a depletion of Lactobacillus spp. in women with IAI although we did not find differences in microbial-diversity. What differentiates our findings from those observed by Hitti *et al*.^11^ was the methodology used to characterize vaginal microbiota composition. The performance of molecular techniques detecting specific bacterial fragments (such as PCR targeted to Lactobacillus spp.) and non-specific bacterial fragments (such as 16S ribosomal RNA gene amplicon sequencing) improves the identification of the composition of vaginal microbiota in comparison to the standard culture used by Hitti *et al*.^11^.

In PPROM without labor, the only previous study describing cervical microbiota in women with IAI was by Kacerovsky *et al*.^12^. These authors observed a significant depletion of *Lactobacillus* spp. and a high abundance of bacteria typically associated with bacterial vaginosis in women exposed to MIAC and/or IAI. In contrast, women with No-MIAC/Non-IAI were characterized by an abundance of *Lactobacillus crispatus* suggesting a protective role of these bacteria against the exposition to MIAC and/or IAI. We found an inverse association between bacterial load of *Lactobacillus* spp. and exposition to IAI. Thus, similar to Kacerovsky *et al*.^12^, we observed a high microbial-diversity in women with IAI with an enrichment of bacteria related to bacterial vaginosis including *Ureaplasma*, *Prevotella*, *Haemophilus* and *Peptoniphilus*. Interestingly, the presence of IAI was related to a depletion of *Lactobacillus* spp. load.

In line with other authors^13–15^, we also observed an inverse association between total *Lactobacillus* genus load and gestational age at delivery. However, unlike some authors such as Kindinger *et al*.^13^ and Petricevic *et al*.^14^, we did not observe an association between *Lactobacillus iners* and the risk of sPTB.

In women with PPROM, Brown *et al*.^20^ did observe an association between vaginal dysbiosis and the occurrence of histological chorioamnionitis and funisitis but Jayaprakas *et al*.^21^ did not. Unfortunately, we were not able to accurately evaluate the association between vaginal microbiota and histological chorioamnionitis or funisitis. However, we found microbial-diversity to be a risk factor (OR 4.07 95% CI 1.2–14.1) and *Lactobacillus* spp., a protective bacteria (OR of 0.5 95% CI 0.29–0.89) for the subsequent occurrence of clinical chorioamnionitis.

One of the main strengths of our study was the well-characterized infectious/inflammatory phenotype of our unique cohort that routinely performs amniocentesis to identify IAI as part of clinical management in women with PTL. Concerning this, there is emerging data emphasizing the importance to initiate antenatal antibiotic treatment early in the group of women with MIAC. Thus, Yoon *et al*.^22^ have recently reported that eradication of MIAC and IAI was possible after broad-spectrum antibiotic treatment in a substantial number of women with PTL. This emphasizes the importance of performing amniocentesis for this indication in women with PTL with early onset of symptoms. Another strength of this study was that we characterized *Lactobacillus* spp. by both 16S ribosomal RNA gene amplicon sequencing and specific quantitative PCR.

Although one of the limitations was the high rate of antibiotics administered in the entire study group (approximately 30% women were treated with antibiotics prior to vaginal sampling), we took into consideration this variable in our regression models. Finally, we acknowledge we did not characterize AF microbiota composition by 16S ribosomal RNA gene amplicon sequencing. However, to identify microorganisms in the AF, we performed not only specific culture media but also Sanger sequencing of the 16S ribosomal RNA gene.

From a clinical perspective, directions for future research should evaluate in larger cohort of women with PTL, the diagnostic performance of vaginal bacterial load of Lactobacillus spp. as a non-invasive marker of the intra-uterine environment.

In conclusion, we observed a reduced *Lactobacillus* load in women exposed to IAI, in those with an early gestational age at delivery or a short latency from vaginal sampling to delivery and found microbial-diversity and *Lactobacillus* spp. depletion to be associated with subsequent clinical chorioamnionitis.

## Methods

### Study design

From an observational cohort study (2008–2016) performed in women with PTL <34 weeks who had undergone amniocentesis to rule out IAI and with vaginal fluid collected, we selected 69 consecutive women. These women were admitted to the Department of Maternal-fetal Medicine at the Hospital Clinic, Barcelona, Spain.

Women with clinical signs of chorioamnionitis at admission, multiple gestation, PPROM, women who did not consent to participate in the study and those in whom amniocentesis was not technically possible were not eligible for this study.

Patient selection and sampling procedures were performed in accordance with the Declaration of Helsinki and applicable local regulatory requirements after approval from the Ethics Committee of the Hospital Clinic of Barcelona (HCB/2010/5811; HCB/2017/0821). Written informed consents were obtained from all subjects.

### Clinical definitions and management

Gestational age was established according to the first-trimester ultrasound scan.

The definition of PTL and antenatal management has been reported previously^23,24^. Briefly, the standard antenatal management of women with PTL included lung maturation with a complete course of antenatal steroids (betamethasone 12-mg intramuscular injection with two doses given 24 hours apart) at between 24.0 to 34.6 weeks. Tocolysis (nifedipine or atosiban) was considered to complete maturation with steroids in the absence of clinical chorioamnionitis, placenta abruption or non-reassuring fetal status. In women with a diagnosis of MIAC tocolysis was discontinued. Regarding antibiotic treatment, women with AF glucose levels <5 mg/dL and/or with microorganisms determined by Gram staining and/or positive cultures were treated with parenteral antibiotics during 10 days according to the antibiogram of the microorganism isolated. Prophylactic antibiotics were also considered (endovenous ampicillin 1 g/6 h, endovenous gentamycin 80 mg/8 h and oral azithromycin 1 g) in women with advanced cervical dilatation (Bishop index ≥6) until AF culture results were obtained. In case of negative cultures, prophylactic antibiotic treatment was discontinued. From 2011 onwards, magnesium sulfate was administered for fetal neuroprotection between 24.0 and 32.0 weeks if imminent labor was suspected.

Clinical chorioamnionitis was defined based on the criteria of Gibbs *et al*.^25^.

### Amniotic fluid and vaginal fluid collection

Amniotic fluid (AF) was obtained by transabdominal amniocentesis at admission and kept at 4 °C until processing.

Vaginal fluid was collected using swabs (Cytobrush Plus GT; Medscan Medical AB) from the posterior vaginal fornix immediately after amniocentesis or within 24 h. Each Cytobrush was submerged in 1.0 mL of sodium chloride (NaCl) (9 mg/mL) and kept at 4 °C until processing. Vaginal fluid was centrifuged at 3000 g at 4 °C for 10 minutes and pellet was stored at −80 °C until analysis.

### Diagnosis of IAI and MIAC

In all samples, we measured the level of IL-6 in AF by enzyme-linked immunoassay (ELISA) (Biosource; Invitrogen, Carlsbad, CA), previously centrifuged at 4,000 rpm for 10 minutes at 4 °C and stored at −80 °C. The minimum detectable level of IL-6 was 0.2 ng/mL. We defined IAI according to the receiver operating characteristic curve analysis of IL-6 (expressed in a log scale) previously employed by our group^4,23,26^ and similar to that reported by Romero *et al*.^27^.

MIAC was defined by the presence of a positive AF culture for bacteria (chocolate agar for aerobes, Schaedler agar for anaerobes and thioglycollate broth) and for *Ureaplasma* spp. or *Mycoplasma hominis* (Mycoplasma IST 2, bioMérieux). All samples were further analyzed by specific polymerase chain reaction (PCR) amplification of the 16S ribosomal RNA gene using the primers: 5′- AGA GTT TGA TCC TGG CTC AG - 3′ and 5′- GGA CTA CCA GGG TAT CTA AT - 3′ followed by Sanger sequencing in the Department of Microbiology. Sequences were identified using the Blast algorithm in the NCBI database, with minimum 98% sequence identity.

### DNA extraction, 16s ribosomal RNA gene amplification and sequencing

DNA extraction from the vaginal swabs was performed using the Purelink Microbiome DNA Purification Kit (Invitrogen) according to the manufacturer’s instructions. DNA concentrations were measured using a Qubit® 2.0 Fluorometer (Life Technology, Carlsbad, CA, USA).

The 16S ribosomal RNA sequencing library was constructed following the 16S rDNA gene Metagenomic Sequencing Library Preparation Illumina protocol, targeting the V3 and V4 hypervariable regions. After 16S rDNA gene amplification, the mutiplexing step was performed using the Nextera XT Index Kit (Illumina, Inc.). The libraries were sequenced using a 2 × 300pb paired-end run (MiSeq Reagent kit v3 (Illumina, Inc.)) on a MiSeq Sequencer according to the manufacturer’s instructions (Illumina, Inc.).

### Data and statistical analysis

Quality assessment was performed with the use of the prinseq-lite program^28^ (min_length: 50; trim_qual_right: 30; trim_qual_type: mean; trim_qual_window: 20). Forward and reverse reads were joined using *FLASH* program^29^ applying default parameters.

Chimeric sequences were removed using *the UCHIME program version 4.2. Open reference OTU picking was performed* at 97% identity and were classified using the RDP algorithm^30^ in combination with the RDP database version 11.

Samples with less than 1,000 sequence reads were removed. Singletons and OTUs with a relative frequency <0.01% were also removed. A maximum of 3,000 taxa were included. Sequences classified as *Cyanobacteria* and/or *Chloroplasts* were removed.

OTU tables were rarefied to 8,054 sequences per sample to avoid variations in sequencing depth. To assess alpha-diversity, richness (Chao1 and ACE indexes) and microbial-diversity (Shannon and Simpson indexes) were computed at genus and OTU levels. Beta-diversity using weighted and unweighted UNIFRAC (phylogenetic distance) and Bray Curtis distance (non-phylogenetic) were used as input for ordination analysis using non-metric multidimensional scaling (NMDS). The Calypso software version 8.24 (http://cgenome.net/calypso/) was used for data mining and multivariate analysis. Linear discriminant analysis effect size (LEfSe) was used to detect unique biomarkers (linear discriminant analysis (LDA) score >3.0) in relative abundance of bacterial taxonomy^31^.

### Bacterial load of *Lactobacillus* spp. by quantitative PCR

Quantitative PCR (qPCR) amplification and detection were performed with specific primers targeted to the 16S region for *Lactobacillus* spp.^32^ and a specific toxin from *Lactobacillus iners*^33^ in each vaginal sample. Each reaction mixture of 20 μl was composed of KAPA Sybr Fast qPCR Kit (KAPA Biosistems), 0.4 μl of each primer (10 μM concentration) and 1 μl of template DNA in a Light Cycler 480 Real-Time PCR System (Roche Technologies). All amplifications were performed in duplicates. The bacterial concentration in each sample was calculated by comparison with the Ct values obtained from a standard curve and also, a negative control was included in each reaction plate. These were generated using serial 10-fold dilutions of gene.

Data was normalized for total DNA concentrations (ng/μL) and presented in a logarithmic scale (log number copies gene/ng total DNA).

### Statistical analysis

Statistical analysis of demographic data and pregnancy outcomes were performed using the SPSS 20.0 for MAC OS (IBM Corporation, USA). The normality of the data was tested using the Kolmogorov-Smirnov normality test and the Shapiro-Wilk test. Continuous variables were presented as median (25^th^, 75^th^ percentile). Categorical variables were presented as numbers or percentages (%). The association of vaginal microbial-diversity and bacterial load of *Lactobacillus spp*. (expressed as log n copies gene/ng total DNA) to predict different outcomes was evaluated using linear and logistic regression analysis. Regression models took into consideration whether antibiotic was administered prior vaginal sampling. Receiver operating characteristics (ROC) curve analysis was employed to display the relationship between sensitivity and the false-positive rate (1 – specificity) of bacterial load of *Lactobacillus* spp. for diagnosing sPTB <34 weeks.

All differences were considered statistically significant with a *p* < 0.05 with two-sided alternative hypotheses.

## Supplementary information

Supplemental S1
