# Supplementary material for: Characterization of vaginal microbiota in women with preterm labor with intra-amniotic inflammation
Source: Sci Rep. 2019 Dec 12;9:18963. doi: 10.1038/s41598-019-55611-y (PMC6908687; doi:10.1038/s41598-019-55611-y)
Supplement: Supplementary file 1 — Supplemental S1 [file 41598_2019_55611_MOESM1_ESM.pdf]

Characterization of vaginal microbiota in women with preterm labor with intra-amniotic inflammation

Teresa Cobo[*], M.D., Ph.D; Andrea Vergara, PharmD; Maria Carmen Collado, Ph.D; Climent Casals-Pascual, M.D., Ph.D; Eduardo Herreros, MSc; Jordi Bosch, M.D., Ph.D; Ana B Sánchez-García, BSc; Rosa López-Parellada, MSc; Júlia Ponce, M.D., Eduard Gratacós, M.D., Ph.D.

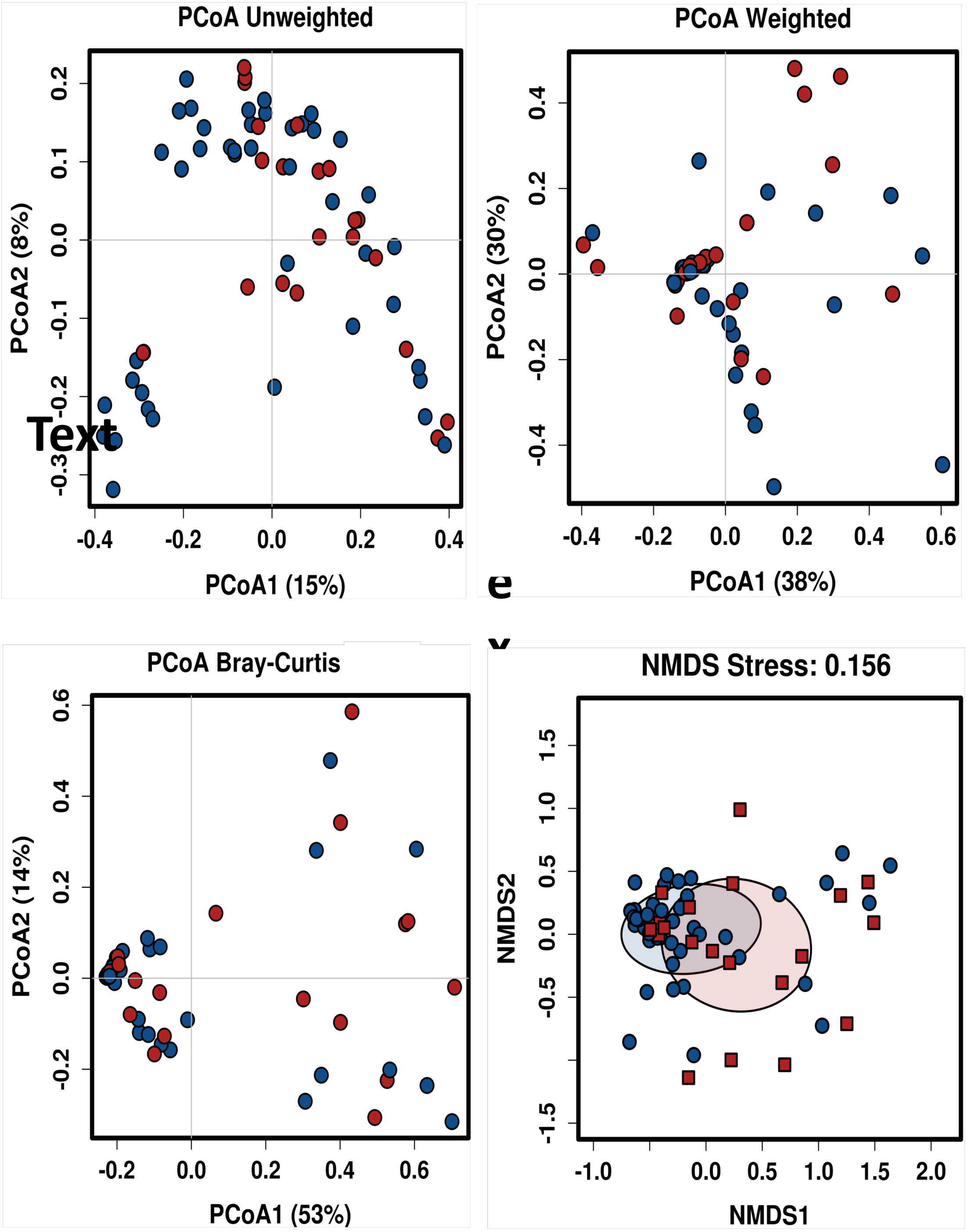
PCoA Unweighted
PCoA2 (8%)
PCoA1 (15%)
-0.4 -0.2 0.0 0.2 0.4
-0.3 -0.2 -0.1 0.0 0.1 0.2
Text
PCoA Weighted
PCoA2 (30%)
PCoA1 (38%)
-0.4 -0.2 0.0 0.2 0.4 0.6
-0.4 -0.2 0.0 0.2 0.4
e
y
PCoA Bray-Curtis
PCoA2 (14%)
PCoA1 (53%)
-0.2 0.0 0.2 0.4 0.6
-0.2 0.0 0.2 0.4 0.6
NMDS Stress: 0.156
NMDS2
NMDS1
-1.0 0.0 0.5 1.0 1.5 2.0
-1.5 -0.5 0.0 0.5 1.0 1.5
